# Diagnosis and Surgical Management of a Retroperitoneal Lipoma in Pregnancy

**DOI:** 10.1155/2020/6309417

**Published:** 2020-07-16

**Authors:** Katharina Mitchell, Kylie Fuller, Alan Thomay, Robert Shapiro

**Affiliations:** West Virginia University Department of General Surgery and Obstetrics and Gynecology, Morgantown WV, USA

## Abstract

Retroperitoneal lipomas during pregnancy are very rare. We report a case of a 29-year-old pregnant female who presented with a retroperitoneal lipoma. Our patient presented at 15-week gestation with abdominal pain, distention, and orthopnea. Due to vague symptoms and nonspecific imaging capabilities, retroperitoneal tumors in pregnancy are uniquely challenging with regard to diagnosis and treatment. We describe the unique work up of a retroperitoneal lipoma in pregnancy and the risks and benefits which were considered when optimizing care to the patient. Percutaneous core needle biopsy has accuracy rates for pathologic diagnosis of up to 98% and is largely safe to perform during pregnancy. Surgical resection of this type of tumor does not mandate cesarean delivery in subsequent pregnancies.

## 1. Introduction

Retroperitoneal tumors in general are rare, making up less than 1% of all neoplasms diagnosed [1]. When found, they often have a malignant transformation, with the most common being liposarcoma [2]. Due to their location, these tumors generally are asymptomatic but can present with nonspecific gastrointestinal complaints [3].

A retroperitoneal tumor diagnosed in pregnancy presents unique challenges given its strong malignant potential. The literature describing retroperitoneal tumors in pregnancy is sparse, and little information is available to help guide surgical management. This case report describes the work up and surgical management of a retroperitoneal tumor originating from the round ligament during pregnancy.

## 2. Case Presentation

**Figure 1:**
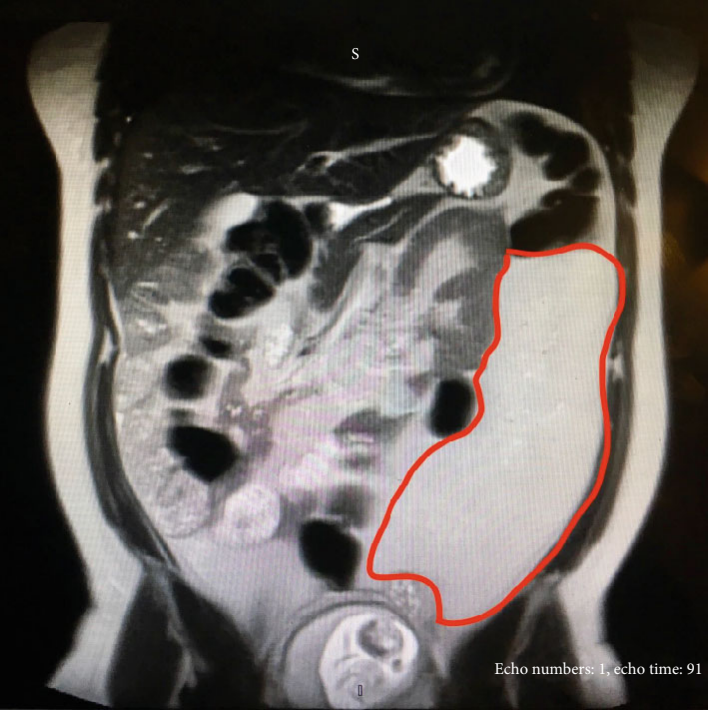
T2-weighted MRI at 15-week gestation demonstrates large homogeneous fat-containing mass which measured 28 × 14 × 6 cm with mass effects of the bowel. A minimal amount of intralesional fluid is noted; no irregular septa are identified, consistent with a simple lipoma.

**Figure 2:**
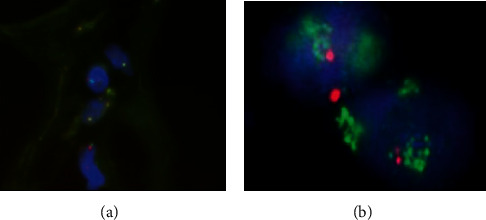
Fluorescence in situ hybridization (FISH) analysis was performed using a dual-color MDM2/CEN 12 probe set to detect gene amplification. MDM2 amplifications (neon green) are frequently detected in well-differentiated liposarcoma. (a) No MDM2 gene amplification is identified by FISH analysis in our patient; (b) amplification of the MDM2 gene in a well-differentiated liposarcoma (fluorescence in situ hybridization, FISH).

**Figure 3:**
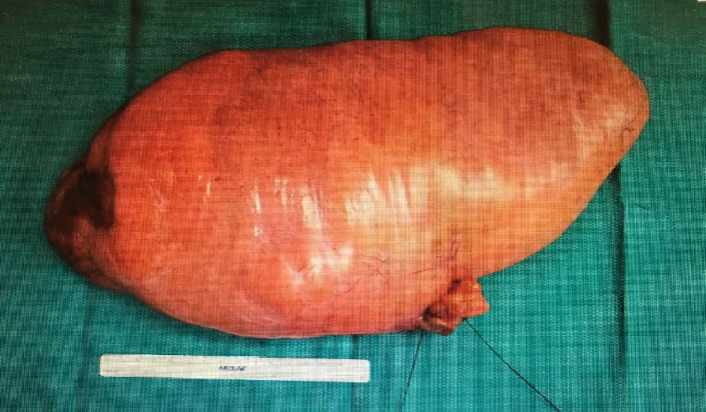
Very large (>25 cm) fatty tumor that was noted in the abdominal cavity to be entirely covered by retroperitoneum and arising from the left pelvic sidewall.

**Figure 4:**
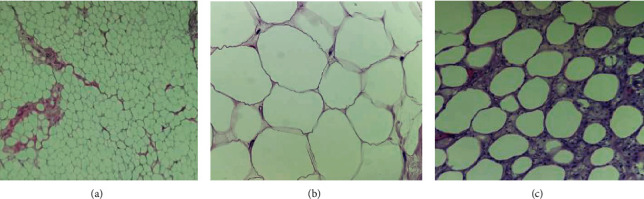
(a) Mature adipocytes that vary little in size from one another. Thin fibrous septa are seen separating the lobules. (b) The adipocyte nucleus is bland, small, and compressed against the periphery of the cell by the large fat vacuole. (c) Area of fat necrosis with foamy histiocytes surrounding and reacting to damaged adipocytes.

The patient is a 29-year-old African-American female with no significant past medical history who presented at 15-weeks gestation with abdominal pain, distention, and orthopnea. The patient denied constipation, weight loss, or nausea. She had an uncomplicated prenatal course. On physical exam, the patient's abdomen was nontender, mildly distended, and asymmetrical in the left upper and lower quadrant. Magnetic Resonance Imaging (MRI) showed a large mass in her left retroperitoneal space measuring 28 × 14 × 6 cm compressing her bowel (Figure 1). The patient underwent a percutaneous needle biopsy which demonstrated mature adipose tissue consistent with a benign lipoma. Gene amplification studies were performed on the biopsy specimen using fluorescence in situ hybridization (FISH) and showed no evidence for malignancy (Figure 2). Due to the benign histopathology and the stable size of the lesion, it was decided surgical resection would take place after delivery. Ten weeks postpartum, the patient underwent exploratory laparotomy with resection of the left pelvic fatty tumor (Figure 3). Frozen section pathology slides confirmed the diagnosis of a retroperitoneal lipoma (Figure 4). The tumor was confined to the retroperitoneum and round ligament without invasion of the other intra-abdominal structures. Final pathology confirmed a mature lipoma.

The patient's postoperative course was uneventful, and she was discharged on postoperative day number four. The patient subsequently developed an umbilical hernia; however; she never had any complications from the hernia apart from abdominal pain which subsided. The patient has been able to conceive again, and the route of delivery was not affected by the previous surgical removal of her retroperitoneal lipoma.

## 3. Discussion

To our knowledge, this is the first reportable case of asymptomatic retroperitoneal lipoma diagnosed in pregnancy. Lipomas from the round ligament consist of herniated fat that originates from the retroperitoneal space outside and posterior to the transverse fascia. It protrudes through the internal ring lateral to the uterus. These tumors can obtain large dimensions due to their location and insidious growth [4].

Retroperitoneal lipomas that occur during pregnancy can be extremely challenging to diagnose and treat. Firstly, the intestinal symptoms of the lipoma can be confused with common symptoms of pregnancy, such as nausea, vomiting, constipation, esophageal reflux, and defecatory dysfunction [5]. Patients with retroperitoneal lipomas can also present with symptoms mimicking appendicitis, which can result in emergent surgery [6]. This is obviously not ideal during pregnancy. Secondly, traditional imaging, such as X-ray and CT, is the diagnostic modality of choice for abdominal masses but is limited in pregnancy due to their potential for teratogenic effects. These include an increased risk of malformations, fetal growth restriction, and miscarriage [4, 7, 8]. Clinicians must then move to second-line imaging choices such as ultrasound and MRI, which may not be as accurate. On MRI, simple lipomas characteristically contain a few thin discrete septa but otherwise are typically homogeneous. Our patient's MRI findings were a large homogeneous fat-containing mass which measured 14 × 6 × 28 cm and caused a mass effect on the bowel (Figure 1). The mass seen contained a minimal amount of fluid and no irregular septa. Thirdly, performing a retroperitoneal abdominal biopsy in pregnancy poses serious risks to the fetus, such as fetal demise and/or early delivery [9]. A study looking at kidney biopsy in pregnancy showed evidence that the risk of biopsy increased with advancing gestational age, reaffirming the need for early diagnosis of abdominal masses in pregnancy [9].

The need for urgent surgery to treat the retroperitoneal lipoma is case specific and depends on a multitude of factors such as symptoms, rate of growth, trimester, and patient preference [10]. Patients must be counseled extensively regarding the risks of surgical intervention during pregnancy. Fortunately, recent advances in work up of abdominal masses in pregnancy can help guide this often very difficult decision.

Percutaneous core needle biopsies, like our patient underwent, have become more common exhibiting accuracy rates of 80% to 98% [11]. Extensive literature exists reaffirming the safety and efficacy of percutaneous breast biopsy during pregnancy regardless of gestational age [12]. There is no reason to believe this literature would not apply to other areas of the body. Our best imaging modalities, such as MRI, have considerably less positive predictive value for malignancy. A recent study demonstrated that 63% of lesions considered suspicious for well-differentiated liposarcoma on MRI were actually benign simple lipomas (13%) and benign lipoma variants (50%) [13].

The prognostic factors of a liposarcoma include tumor size, grade, location, surgical resection, and histological subtypes. Pleomorphic liposarcomas and dedifferentiated tumors are associated with a poor prognosis due to their highly aggressive nature with higher rates of metastasis and recurrence [13]. Therefore, pathologic confirmation is essential so as not to delay treatment [14]. In this case, after a risk-benefit discussion with the patient, the decision was made to biopsy the mass, which demonstrated benign mature adipose tissue.

The definitive management of retroperitoneal lipomas is commonly surgical, which is generally not difficult due to the capsule that surrounds the lipoma, creating a good surgical plane [4]. When malignancy is less likely, most surgical procedures in pregnant patients are delayed until the postpartum period in order to prevent secondary harm to the fetus [15]. In a study on surgical resection of ovarian tumors during pregnancy, the risk of miscarriage following both laparotomy and laparoscopy was between 3% and 5% [16].

When surgical management is the best course of action, like in cases where the biopsy demonstrates malignancy or the patient is experiencing symptoms, the American College of Obstetrics and Gynecology (ACOG) recommends delaying surgery until at least the second trimester, when contractions and spontaneous abortion are considerably less likely [15]. In our patient, due to the manageable symptoms and the benign nature of the tumor, the decision was made to postpone the operation until the postpartum period.

## 4. Conclusions

1. Due to vague symptoms and nonspecific imaging capabilities, retroperitoneal tumors in pregnancy are uniquely challenging with regard to diagnosis and treatment
2. Percutaneous core needle biopsy has accuracy rates for pathologic diagnosis of up to 98% and is largely safe to perform during pregnancy
3. Surgical resection of this type of tumor does not mandate cesarean delivery in subsequent pregnancies

## References

[1] Wei D., Shen L., Yang K., Fang F. (2013). Giant retroperitoneal lipoma in a pregnant patient. *Journal of Obstetrics and Gynaecology*.

[2] Weniger M., D’Haese J. G., Kunz W. (2015). En-bloc resection of a giant retroperitoneal lipoma: a case report and review of the literature. *BMC Research Notes*.

[3] Chaudhry S. R., Chaudhry K. (2019). *Anatomy, abdomen and pelvis, uterus round ligament. StatPearls. Treasure Island (FL): StatPearls Publishing*.

[4] Mathen S., Nockolds C., Arutchelvam S. (2014). Large intraperitoneal lipoma in pregnancy. *BML Case Reports*.

[5] Davis D. C. (1996). The discomforts of pregnancy. *Journal of Obstetric, Gynecologic, and Neonatal Nursing*.

[6] Miller T. J., Paulk D. G. (2013). Round ligament lipoma mimicking acute appendicitis in a 24-week pregnant female: a case report. *Hernia*.

[7] DiSantis D. J., Ralls P. W., Balfe D. M., Bree R. L., Glick S. N., Kidd R. (2000). Imaging evaluation of the palpable abdominal mass. American College of Radiology. ACR Appropriateness Criteria. *Radiology*.

[8] Patel S. J., Reede D. L., Katz D. S., Subramaniam R., Amorosa J. K. (2007). Imaging the pregnant patient for nonobstetric conditions: algorithms and radiation dose considerations. *Radiographics*.

[9] Piccoli G. B., Daidola G., Attini R. (2013). Kidney biopsy in pregnancy: evidence for counselling? A systematic narrative review. *BJOG : An International Journal of Obstetrics and Gynaecology*.

[10] Huo D., Liu L., Tang Y. (2015). Giant retroperitoneal liposarcoma during pregnancy: a case report. *World Journal of Surgical Oncology*.

[11] Walker J. B., Stockwell E., Worhacz K., Kang P., Decomas A. (2018). Safety and accuracy of core needle biopsy for soft tissue masses in an ambulatory setting. *Sarcoma*.

[12] Collins J. C., Liao S., Wile A. G. (1995). Surgical management of breast masses in pregnant women. *The Journal of Reproductive Medicine*.

[13] Gaskin C. M., Helms C. A. (2004). Lipomas, lipoma variants, and well-differentiated liposarcomas (atypical lipomas): results of MRI evaluations of 126 consecutive fatty masses. *AJR. American Journal of Roentgenology*.

[14] Lilly M. C., Arregui M. E. (2002). Lipomas of the cord and round ligament. *Annals of Surgery*.

[15] OʼShea M. (2018). Nonobstetric surgery during pregnancy. *Obstetrics and Gynecology*.

[16] Berczi C., Osvath P., Flasko T. (2015). Large benign retroperitoneal tumour in pregnancy. *Canadian Urological Association Journal*.

